# Prediction of sentinel lymph node metastasis in breast cancer patients based on preoperative features: a deep machine learning approach

**DOI:** 10.1038/s41598-024-51244-y

**Published:** 2024-01-16

**Authors:** Reza Shahriarirad, Seyed Mostafa Meshkati Yazd, Ramin Fathian, Mohammadmehdi Fallahi, Zahra Ghadiani, Nahid Nafissi

**Affiliations:** 1 Thoracic and Vascular Surgery Research Center, Shiraz University of Medical Science, Shiraz, Iran; 2 Department of Surgery, Tehran University of Medical Sciences, Tehran, Iran; 3 Faculty of Engineering, University of Alberta, Edmonton, AB Canada; 4 School of Medicine, Jahrom University of Medical Sciences, Shiraz, Iran; 5 Department of Breast, Rasoul Akram Hospital Clinical Research Development Center (RCRDC), Iran University of Medical Sciences, Tehran, Iran

**Keywords:** Outcomes research, Surgical oncology, Machine learning, Predictive medicine, Software, Breast cancer

## Abstract

Sentinel lymph node (SLN) biopsy is the standard surgical approach to detect lymph node metastasis in breast cancer. Machine learning is a novel tool that provides better accuracy for predicting positive SLN involvement in breast cancer patients. This study obtained data from 2890 surgical cases of breast cancer patients from two referral hospitals in Iran from 2000 to 2021. Patients whose SLN involvement status was identified were included in our study. The dataset consisted of preoperative features, including patient features, gestational factors, laboratory data, and tumoral features. In this study, TabNet, an end-to-end deep learning model, was proposed to predict SLN involvement in breast cancer patients. We compared the accuracy of our model with results from logistic regression analysis. A total of 1832 patients with an average age of 51 ± 12 years were included in our study, of which 697 (25.5%) had SLN involvement. On average, the TabNet model achieved an accuracy of 75%, precision of 81%, specificity of 70%, sensitivity of 87%, and AUC of 0.74, while the logistic model demonstrated an accuracy of 70%, precision of 73%, specificity of 65%, sensitivity of 79%, F1 score of 73%, and AUC of 0.70 in predicting the SLN involvement in patients. Vascular invasion, tumor size, core needle biopsy pathology, age, and FH had the most contributions to the TabNet model. The TabNet model outperformed the logistic regression model in all metrics, indicating that it is more effective in predicting SLN involvement in breast cancer patients based on preoperative data.

## Introduction

Breast cancer is the most commonly diagnosed type of cancer, accounting for 11.7% of all cancer sites and an estimated number of 4.1 million cases in the US by 2022, and a prevalence of 23.6% among women in Iran. Furthermore, with a mortality rate of 15.5%, this cancer is the leading cause of cancer death in women worldwide^1–3^. Lymphatic drainage of the breast plays an essential role in spreading cancerous cells and metastasis to distant organs^4^. The most common draining node field from all breast regions is the axillary node field, with an overall probability of 98.2%, which makes these nodes a significant prognostic factor for cancer staging and management^5,6^.

Axillary lymph node dissection (ALND) was proposed by Halsted et al. in 1898 as a radical approach. It had been performed in addition to mastectomy on all primary breast cancer patients for decades^7,8^. In the past few decades, the high rate of morbidity in patients and the need for a less invasive method led to the introduction of Sentinel Lymph Node biopsy (SLNB), which has a similar 10-year survival and tumor recurrence in breast cancer patients as an initial alternative to ALND^9–13^.

Sentinel Lymph nodes (SLNs) are the first lymphatic nodes that receive metastatic deposits of cancerous cells. These nodes are localized using radioisotope, blue dye, or both, and a subsequent biopsy is performed on marked lymph nodes to evaluate metastasis and indicate the next steps^14^. Current guidelines suggest that the biopsy of SLNs must be identified in candidates using appropriate mapping techniques and proceed to ALND if specific criteria are met^15^.

Although SLNB is an advantageous prognostic and diagnostic method, it is still invasive. This procedure, which results in low but present morbidity, heavily depends on the surgeon’s skill and expertise^16–18^. Also, studies showed more than a 10% false negative rate in lymph-positive patients after preoperative systemic therapy^19–21^. Accurate prediction of SLN involvement is essential in helping physicians make informed treatment decisions. Studies evaluated several factors and their relevance in predicting SLN involvement in breast cancer patients^22–27^. Despite advances observed in the literature, accurately predicting SLN involvement remains challenging due to the condition’s complexity and the lack of adequate interpretation and data analysis. Conventional data-driven prediction methods have been proposed in nomogram design models and based on a combination of risk factors^27^. Yet, the generalizability and reliability of these proposed methods have been questioned due to the small sample size and lack of proper validation^28,29^.

Since the past decade, machine learning has illustrated great success by providing high levels of accuracy, precision, and sensitivity in various medical fields with structured data, such as medical images, audio, and text^30–32^. Unstructured medical data, and unstructured data in general, despite being the most common type of data, has yet to see success in achieving the optimal level of accuracy. Recent studies have proposed novel models with high performance while interpretable for unstructured data. One of these models is TabNet, a high-performance and interpretable deep learning architecture for tabular data^32^. TabNet outperformed other state-of-the-art methods for tabular data regarding accuracy and efficiency^33^.

SLN involvement is an important prognostic indicator of breast cancer and can help physicians determine the stage of the disease and make informed treatment decisions. Therefore, the need for a reliable and accurate model to predict SLN involvement which can prevent the morbidity of invasive procedures and efficiently decrease the burden of breast cancer, is evident. On the other hand, the application of machine learning in clinical practice has shown promising results and has been used in other models for lymph node metastasis prediction^34–36^.

The objectives of this research were to: 1. Present the data of 2890 surgical cases of breast cancer patients and conduct a descriptive study to describe demographic and clinical features of surgical breast cancer patients based on sentinel lymph node involvement, 2. Develop a TabNet model, an end-to-end deep learning model, to explore the validity of employing the TabNet model to predict SLN involvement in breast cancer patients undergoing surgery based on the patient’s preoperative clinicopathological factors and compare our model accuracy, specificity, and sensitivity in predicting the SLN involvement using a center-based dataset. We also compared our proposed model’s accuracy, specificity, and sensitivity against the ones from logistic regression analysis. Implementing this method has the potential to revolutionize predictions where the primary form of the data collection is in a tabular format, which in our case is the prediction of SLN metastasis in breast cancer patients based on preoperative features.

## Materials and method

### Study design and data collection

For the first time, we present the dataset of 2890 surgical cases of breast cancer patients obtained from patients with breast tumors referring to two major referral hospitals, Rasoul Akram Hospital and Khatam Al-Anbia Hospital, affiliated with the Iran University of Medical Science and located in Tehran, Iran, during a 22-year period (2000–2021). The dataset consisted of preoperative features, including patient features such as age, family history of breast cancer, gestational factors including first gestational, lactating age, abortion and the number of children, laboratory data including estrogen and progesterone receptor, biomarkers KI67, and also tumoral features such as stage, core needle biopsy (CNB) results, histology, multicenter involvement, size, lymphovascular involvement, and Ductal carcinoma in situ (DCIS) percentage. The mentioned data were collected after obtaining patients’ history, clinical examination, biopsy of the SLN, and histopathological examination. The inclusion criteria for our study were all breast cancer patients during the mentioned period who underwent SLN evaluation. All variables incorporated in the model were based on the data obtained preoperatively; consequently, postoperative indicators, including pathological TNM stage, histological grade, and outcomes, were not included.

The data has been retained adhering to the principles of the Helsinki Convention and the ethics committee of Iran University of Medical Sciences at all stages by the researcher.

### Statistical analysis

The preliminary and baseline results are presented as mean, median, and dispersion, such as the continuous variables are presented as mean ± standard deviation (SD) and ordinal data present median [interquantile range (IQR)] and categorical data present frequency (percent). In order to investigate the statistical relationship between the variables and the sentinel lymph node involvement and the significance of this relationship, the Chi-square test and Fisher’s exact test were performed using two cross-tabulated statistical analyses and the desired parameters to accept or reject the hypothesis of statistical relationship between variables. Additionally, multivariate logistic regression was performed using variables that showed statistical significance (*p*-value < 0.25) to evaluate the prediction properties for SLN involvement. An ultimate *p*-value of less than 0.05 in the logistic regression model was considered statistically significant. IBM SPSS Statistics (Chicago, IL, USA—Version 28, 2018) was used for the statistical analysis of data.

### Data preprocessing and model development

This study proposed TabNet, an end-to-end deep learning model, to predict SLN involvement in breast cancer patients^33^. TabNet encoder includes multiple steps; in the first step, the raw features go through batch normalization. Then, the batch normalized features pass into the feature transformer block. The masks were obtained using the attentive transformer block that employed Sparse feature selection using sparse-matrix. The feature transformer block consists of an n-number (4 for our case) of gated linear unit (GLU) blocks consisting of a fully connected layer, followed by batch normalization and GLU, and the attentive transformer block consists of a fully connected layer, followed by batch normalization layer, prior scales layer, and Sparsemax layer. The prior scales layer contains information about how much each feature has been used previously (at the current decision step). The TabNet model’s learning process was optimized using the Adam optimizer^37^ with a learning rate of 0.02 and a batch size of 256 with Sparsemax as a masking function to select the features^33^. The width of the decision layer and attention embedding for the mask was set to 8, the coefficient for feature reusage in the masks was set to 0.8, the momentum value of 0.3 for the batch normalization, and the gradient values were clipped at 2, and the extra sparsity loss coefficient was 0.0004^33^.

Additionally, balanced accuracy was used as the evaluation metric. The training process was continued for 100 epochs and the best iteration was used for the model. The PyTorch implementation of TabNet (Version 4.0, released on Sep 14, 2022) and Scikit-learn^38^ framework were used to implement the TabNet model and design the training, validation, and test pipeline. Additionally, logistic regression was used as a baseline for this study and compared with the results obtained from the TabNet model.

The dataset included preoperative patient data, laboratory results, tumor features, and gestational factors. The preoperative clinical data was preprocessed to address the missing values and balance the dataset. Then, the data were randomized and missing data points were handled by k-Nearest Neighbors (KNN) imputer. In the next step, the data were undersampled to be balanced. The processed data were split into training and test sets using the leave-one-out cross-validation approach, and the TabNet model was inputted with the labeled data according to postoperative indicators. Ten-fold cross-validation was implemented, with one fold in the test set and the rest in the training set (Supplementary Fig. 1). We evaluated the performance of the TabNet model and compared it with Logistic regression as a base method using F1-score, accuracy, sensitivity, specificity, and the area under the receiver operating characteristic curve (AUC).

### Ethical approval and consent to participate

The present study was approved by the medical ethics committee of the Tehran University of Medical Sciences. Based on the retrospective nature of the study, written informed consent was waived by the Ethics committee of the Iran University of Medical Sciences. Permission to carry out the study and access patient records was sought from the Iran University of Medical Science administrators, and the study was conducted in compliance in accordance with the relevant guidelines and regulations and the Declaration of Helsinki and was also approved by the ethics committee of the university.

## Results

During the 22-year period of our study, a total of 2890 patients with breast cancer were recorded. Among them, 897 (32.9%) were excluded due to no lymphoscintigraphy evaluation and no information regarding SLN status. Among the remaining 1832 patients, 697 (38.0%) had SLN involvement, while 1135 (62.0%) had no involvement. Table 1 demonstrates the clinical and demographic features of our patients.

**Table 1:** Demographical and clinical features of surgical breast cancer patients based on sentinel lymph node involvement.

Variable	Total* (N = 1832)	Sentinel lymph node*		*p*-value**
		Free; n = 1135	Positive; n = 697	
Age (years)	50.90 ± 11.55	51.32 ± 11.70	50.22 ± 11.28	**0.048**
Age group (years)				
≤ 20	1 (0.1)	1 (100)	0 (0)	0.097
20–40	354 (19.3)	204 (57.6)	150 (42.4)	
41–60	1094 (59.7)	678 (62.0)	416 (38.0)	
61–80	382 (20.9)	251 (22.1)	131 (34.3)	
Family history				
Positive	419 (26.2)	280 (66.8)	139 (33.2)	**0.009**
Negative	1180 (73.8)	704 (59.6)	477 (40.4)	
Vascular involvement				
Positive	536 (29.4)	156 (29.1)	380 (70.9)	** < 0.001**
Negative	1290 (70.6)	977 (75.7)	313 (24.3)	
Estrogen receptor				
Positive	1292 (74.3)	790 (61.1)	502 (38.9)	**0.043**
Negative	448 (25.7)	298 (66.5)	150 (33.5)	
Progesterone receptor				
Positive	1249 (71.3)	746 (60.2)	494 (39.8)	**0.001**
Negative	500 (28.7)	343 (68.6)	157 (31.4)	
HER2				
Positive	280 (16.4)	167 (59.6)	113 (40.4)	0.302
Negative	1429 (83.6)	899 (62.9)	530 (37.1)	
Ki67 Percentage (%)	20 [25]	20 [25]	20 [25]	**0.017**
Ki67 Group (%)				
< 20	623 (46.6)	428 (68.7)	195 (31.3)	**0.002**
≥ 20	713 (53.4)	433 (60.7)	280 (39.3)	
Histology				
DCIS	108 (5.9)	104 (96.3)	4 (3.7)	** < 0.001**
IDC	1379 (75.3)	829 (60.1)	550 (39.9)	
Invasive lobular carcinoma	206 (11.2)	126 (61.2)	80 (38.8)	
Mixed ductal lobular carcinoma	96 (5.2)	48 (50.0)	48 (50.0)	
Other	43 (2.3)	28 (65.1)	15 (34.9)	
Core needle biopsy				
Positive	1406 (92.2)	839 (59.7)	567 (40.3)	0.072
Negative	119 (7.8)	81 (68.1)	38 (31.9)	
Side				
Unilateral	1819 (99.5)	1128 (62.0)	692 (38.0)	1.000
Bilateral	10 (0.5)	6 (60.0)	4 (40.0)	
Number of children				
0	131 (9.0)	88 (67.2)	43 (32.8)	0.091
1–3	1007 (69.4)	645 (64.1)	362 (35.9)	
> 3	312 (21.5)	181 (58.0)	131 (42.0)	
First pregnancy age				
≤ 20	720 (50.6)	452 (62.8)	268 (37.2)	0.582
21–30	634 (44.6)	400 (63.1)	234 (36.9)	
> 30	68 (4.8)	47 (69.1)	21 (30.9)	
Lactation (year)				
Till one year	226 (15.8)	129 (57.1)	97 (42.9)	0.118
> 1	1068 (74.5)	686 (64.2)	382 (35.8)	
None	139 (9.7)	90 (64.7)	49 (35.3)	
Oral contraceptive pill use (years)				
None	1012 (70.8)	633 (62.5)	379 (37.5)	0.313
< 5	239 (16.7)	160 (66.9)	79 (33.1)	
≥ 5	178 (12.5)	107 (60.1)	71 (39.9)	
Abortion				
None	977 (69.3)	627 (64.2)	350 (35.8)	0.250
Positive	433 (30.7)	264 (61.0)	9.0)	

*Values are presented as n (%), mean ± Standard deviation, or Median [Interquantile range].

**Bold values indicate a significant association (with a significance level of 0.05).

### Performance of TabNet and logistic regression model

**Figure 1:**
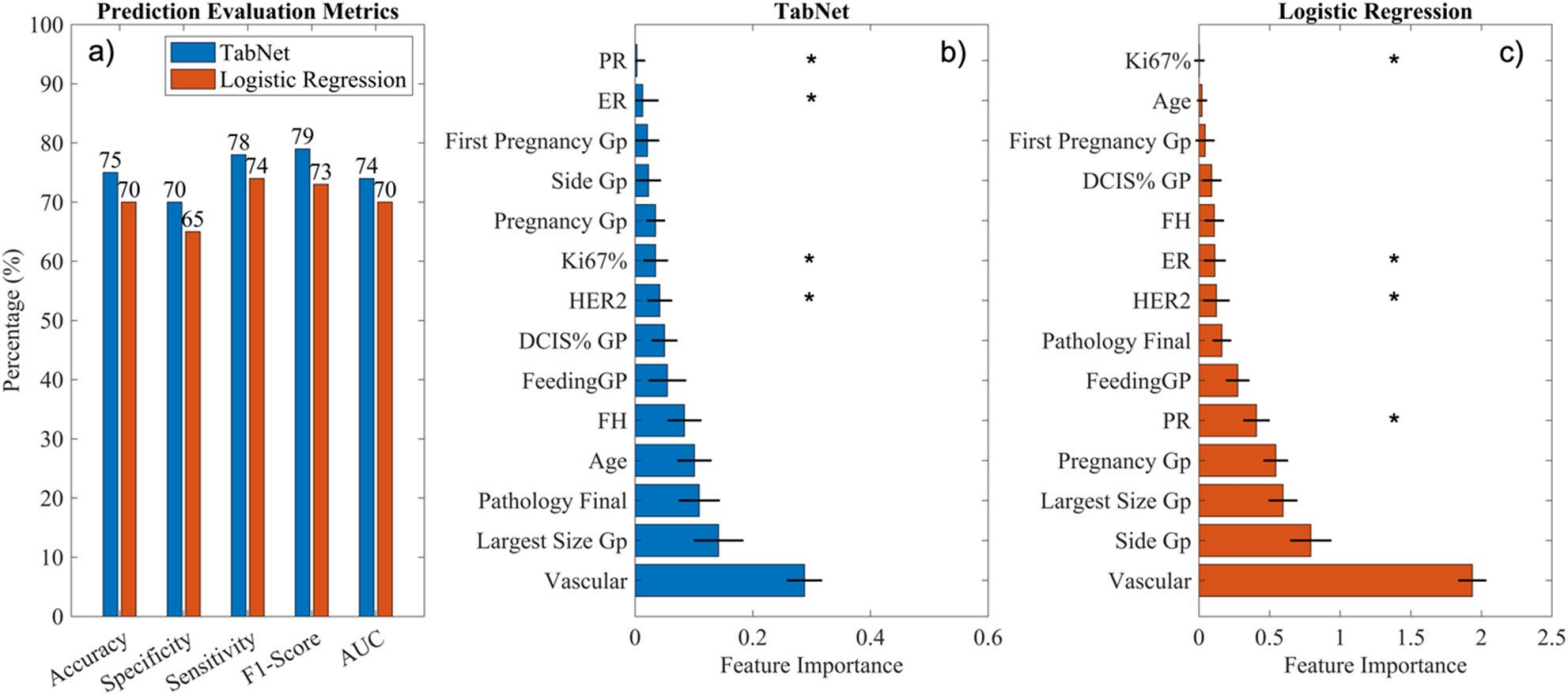
(**a**) Performance comparison between TabNet and Logistic Regression, (**b**) feature importance obtained from the TabNet model, and (**c**) absolute value of variables (features) coefficients represented as feature importance in the logistic model. “*” indicates a significant association (with a significance level of 0.05) between the variable and SLN status. Final Pathology was based on core needle biopsy results. *DCIS* Ductal carcinoma in situ; *ER* Estrogen Receptor; *FH* Family history; *Gp* Group; *PR* Progesterone receptor.

In total, SLN involvement in 1832 breast cancer cases was used to train and validate the model. On average, the TabNet model achieved an accuracy of 75%, precision of 81%, specificity of 70%, sensitivity of 87%, and AUC of 0.74 on the data set, while the logistic regression model demonstrated an accuracy of 70%, precision of 73%, specificity of 65%, sensitivity of 79%, F1 score of 73%, and AUC of 0.70 on the data set (Fig. 1, Supplementary Table S1). Overall, the TabNet model outperformed the logistic regression model in all metrics, indicating that it is a more effective tool for predicting SLN involvement in breast cancer patients. The vascular invasion parameter had the most contribution to the SNL involvement prediction using TabNet model, followed by the tumor’s size, CNB pathology finding, age, and FH (Fig. 1, Supplementary Table S2). In addition to the evaluation metrics, the feature importance that explains how the models reached their predictions was presented for TabNet and the logistic regression model. Feature importance gives understandable feature attributions in the model and increases model explainability. In the logistic regression model, the vascular invasion, unilateral or bilateral, tumor size, first pregnancy age, and PR were the five most contributed features in the SNL involvement prediction (Fig. 1, Supplementary Table S3).

## Discussion

Based on the high impact of SLN involvement in the management and prognosis of breast cancer patients, we proposed a machine learning approach to predict the involvement of this node based on patients’ preoperative features. We achieved a satisfactory predicting capacity for SLN involvement in 1832 breast cancer patients based on preoperative data through the TabNet model.

Our model is the first study to successfully use preoperative tabulated data to predict SLN in breast cancer patients with high accuracy, specificity, and sensitivity. In a study by Fanizzi et al., the authors evaluated SLN metastasis based on histopathological features, by utilizing the logistic regression, Random Forest, and Naïve Bayesian models, and achieved an AUC of 71.5%, 68.1%, and 70.8%, and accuracy of 67.9%, 67.7%, and 66.3%, respectively. Based on their low AUC, and also since logistic regression analysis overruled the other methods, their results were inconclusive and did not support an instrument suitable for actual clinical application^39^. The authors of the mentioned study also demonstrated the incapability of the CancerMath algorithm in detecting SLN metastasis based on clinicopathological features^35^. This was not the case in our study, in which the TabNet model demonstrated superiority compared to the logistic model in terms of SLN prediction. In another study, Liu et al. achieved an AUC of 0.801 and an accuracy of 70.3% using the Bagged-Tree algorithm which does not require feature normalization and is able to reduce the impact of data imbalance. However, their study included features that are mainly obtained in the postoperative period^40^. Our study used the TabNet model and achieved an accuracy of 75%, sensitivity of 78%, specificity of 70%, F1-Score of 79%, and AUC of 0.74 on the test set. To date, our model has demonstrated the highest performance among all SLN prediction methods and is based on baseline preoperative features and CNB results.

Studies focusing on risk and correlating factors with a positive SLN have mostly utilized nomograms and regression analysis^41^. Logistic regression models have a linear nature and are suitable for evaluating the statistical significance of the coefficients in the model^42^. However, these studies carry limitations, such as inferior discrimination in different populations, which could be bypassed with machine learning methods. Although the application of machine learning models in the context of surgical oncology of the breast has been previously reported, our study is the first study with promising AUC and accuracy in predicting SLN metastasis based on preoperative features.

Previous models for SLN prediction among breast cancer patients have mostly focused on imaging and radiological features, such as applying a convolutional neural network (CNN) along with transfer learning on computed tomography (CT) scans, demonstrating an AUC of 0.80 in the primary cohort and 0.82 in the validation cohort while applying deep features extracted from diffusion weighted (DWI) magnetic resonance imaging (MRI) demonstrated an AUC of 0.85 in a test set^43^. However, CT and MRI scans are time-consuming, high-cost, and accompanied by substantial radiation exposure for patients, limiting their application. Zhao et al. utilized three CNN models of deep learning, Inception V3, Inception-ResNet V2, and ResNet-101, to detect axillary lymph node metastasis in breast cancer patients through ultrasound images, which achieved an AUC of 0.89, 0.88, and 0.86, respectively, in predicting lymph node metastasis. A consensus of five radiologists also evaluated their dataset and achieved an AUC of 0.89, 73% sensitivity and 63% specificity from achieved, with a sensitivity of 85% and specificity of 73%; Although this study was applied based on radiotide features and included all lymph nodes, and not specifically SLNs, all their models’ outcome outperformed experienced radiologists, demonstrating the promising role of deep learning models in the detection of metastatic lymph nodes^44^. However, ultrasound in clinical practice is still an operator-dependent technique and is accompanied by procedural limitations^45^.

On the other hand, many models have been developed for subsequent management, treatments, and prognosis after confirming SLN metastasis. In predicting the nodal stage N2-3 after a positive SLNB, the XGBoost model demonstrated satisfactory results and was superior to the logistic model for prediction of the nodal stage N2-3 after a positive SLNB, while the support vector classifier (SVC) model did not reach such accuracy and was lower than the logistic model^41,46^. The SVC by scikit-learn is another model used which builds optimal separating boundaries between data sets and produces dichotomous results^42,47^. This method and the artificial neural network method have been shown to be effective in predicting non-SLN status in SLN-positive breast cancer patients^48,49^. Sugimoto et al. demonstrated the efficacy of an alternative decision tree (ADTree) prediction model to predict axillary lymph node metastasis and response to neoadjuvant chemotherapy in primary breast cancer patients^50^. All these models have potential applications in clinical practice but can be applied following a positive SLNB, which is where our model comes in to provide a prediction of this entity.

The findings of this study indicate that TabNet is a promising tool for predicting SLN involvement in breast cancer patients, benefiting clinicians in making treatment decisions and improving patient outcomes. Following a positive SLNB, surgeons should decide how to approach the potential residual tumor burden of the axilla by carrying out ALND, adjuvant radiotherapy and initiating additional systemic therapies.

Based on the idea of noninvasive prediction, many studies have attempted to use clinical predictors to establish mathematical models to assess the likelihood of SLN metastasis, in which the most practical and efficient predictive models are being developed. The prediction results obtained with the help of a predictive model are more credible than simple clinical guesses. Based on other studies, in the evaluation of features based on nomogram, the correlation between tumor size, tumor location, lymphovascular invasion, and SLN metastasis has been reflected in many models, while the influence of age of onset, histological grade, Ki-67, molecular markers on SLN metastasis has not been unified^24,51–53^. In addition, most of the published models showed relatively unsatisfied discrimination, presenting an AUC lower than 0.7, which was not optimal for guiding clinical practice^24,51–53^. Moreover, some pathological parameters used were postoperative, which limited the clinical application for SLN noninvasive prediction before operation.

In our TabNet model, vascular invasion, tumor size, CNB pathology finding, age, and FH had the highest correlation with SLN involvement, while in the logistic regression model pathology results, age, and FH were replaced with unilateral or bilateral involvement of tumor, first pregnancy age, and PR. Viale et al., Bevilacqua et al., and Veerapong et al. found vascular invasion and pathologic histology to be significantly associated with positive SLNB using their logistic regression models^22,24,25^. Ding et al. only found histological grade, tumor size, and age as independent predictors for SLN metastasis. However, they mentioned limitations in evaluating lymphovascular invasion through CNB^54^. Different histotypes of breast cancer have different potentials for metastasis, and lymphovascular structures are the path for cancerous cells to reach the lymph nodes, which can explain the high association of these factors and SLN metastasis.

Pregnancy, and lactation were interesting factors that were significantly associated with SLN status, and Hassan et al. showed the same association with the former using a support vector machine model to predict SLN status in elderly patients^36^. Pregnancy was shown to be associated with a lower chance of luminal breast cancer^55^. These factors are important because they are applicable in outpatient settings and can be used for screening and easy risk evaluation.

Another important factor to include in screening and outpatient settings is age. We found a significant association between age and SLN status using our model, as most previous studies did by using regression models and the support vector machine model by Hassan et al., all proposing an inverse correlation between age and probability of positive SLNB^24–26,36,54^. Viale et al. did not find this factor significantly associated with their logistic regression model^22^. Breast cancer tends to be more aggressive in younger patients, which could cause a significant association between young age and positive SLNB^56^.

Progesterone, estrogen receptors, and HER-2 are important biomarkers in breast cancer classification, and we found a significant association between the two latter and SLN metastasis. Viale et al., Bevilacqua et al., and Hassan et al. proposed the same association between progesterone receptor status and SLN metastasis, and Ceylan et al. proposed HER-2 status association with SLN metastasis. Bevilacqua et al. and Hassan et al. also proposed the association of estrogen receptor status^22,24,27,36^. All these studies used logistic regression analysis to develop their model, except Hassan et al. model, which was developed using a support vector machine. Further studies in larger populations using different novel methods are needed to overcome this heterogeneity in outcomes.

One of the strengths of this study is the use of a large and diverse dataset. The dataset included a variety and large scale of patient and tumor characteristics, providing a realistic representation of the population. While this allowed for consistent data collection, it may not be generalizable to other populations. Future research should aim to evaluate the performance of TabNet in larger and more diverse datasets to confirm its effectiveness in different populations. Another topic that should be considered when evaluating the generalizability is the missing values in the dataset, which was inevitable based on the retrospective nature. For our study, patients with missing data were not excluded (to simulate real clinical setting) and KNN imputer was used to handle the missing values. Consequently, the ability of our proposed method (TabNet) was investigated and compared against the logistic regression model. Not excluding cases with missing data and using KNN imputer might have impacted the performance of the TabNet and logistic regression model and higher performance could have been achieved with a better data set or alternative imputer. However, we believe that our model can be incorporated into future prospective studies to confirm the realistic performance of the model and overcome the potential bias derived from retrospective data. Furthermore, subsequent studies can include more diversified breast cancers and also multiple national and international centers to generate a real-world distribution of patients to better train the TabNet for improved stability. Despite these limitations, the results of this study provide valuable insights into the effectiveness of TabNet in predicting SLN involvement in breast cancer patients. Its ability to accurately predict SLN involvement can aid in making treatment decisions and improving patient outcomes. Future studies could investigate the use of custom loss and learning rate annealing on the overall performance of the model. Considering the availability of CT and MRI images, another venue for investigation would be integrating the imaging data with the tabular data, which should be possible by customizing the TabNet model.

## Conclusion

The aim of this paper is to investigate the potential use of deep learning models (in our case TabNet) for predicting SLN involvement in breast cancer patients and compare the outcome of the TabNet model with the more conventional methods available in the literature. In conclusion, the use of TabNet for predicting SLN involvement in breast cancer patients has several potential advantages, including its ability to provide more accurate predictions, make predictions in real-time, and reduce the need for manual data analysis and interpretation. However, there are also some limitations to the use of TabNet, including the potential lack of generalizability that could be investigated by having a more extensive and diverse dataset.

### Supplementary Information

Supplementary Information.

## Data Availability

The datasets used and/or analyzed during the current study are available from the corresponding author on reasonable request and with permission of the Research Ethics Committee of Iran University of Medical Sciences.
