# Supplementary material for: Prediction of sentinel lymph node metastasis in breast cancer patients based on preoperative features: a deep machine learning approach
Source: Sci Rep. 2024 Jan 16;14:1351. doi: 10.1038/s41598-024-51244-y (PMC10791698; doi:10.1038/s41598-024-51244-y)
Supplement: Supplementary file 1 — Supplementary Information. [file 41598_2024_51244_MOESM1_ESM.docx]

**Supplementary Materials**

**Table S1.** Evaluation metric of TabNet vs Logistic Regression Model

| **Values** | **TabNet Model** | | **Logistic Regression Model** | |
| --- | --- | --- | --- | --- |
|  | Average (%) | SD (%) | Average (%) | SD (%) |
| Accuracy | **75%** | 4% | 70% | 3% |
| Precision | **81%** | 3% | 73% | 2% |
| Specificity | **70%** | 3% | 65% | 2% |
| Sensitivity | **78%** | 8% | 74% | 2% |
| F1-Score | **79%** | 5% | 73% | 2% |
| AUC | **0.74** | 0.03 | 0.70 | 0.03 |

AUC: area under the curve; SD: standard deviation

**Table S2.** Feature Importance during each fold for TabNet Model (sorted largest to smallest)

|  | Fold 1 | Fold 2 | Fold 3 | Fold 4 | Fold 5 | Fold 6 | Fold 7 | Fold 8 | Fold 9 | Fold 10 | Average | STD |
| --- | --- | --- | --- | --- | --- | --- | --- | --- | --- | --- | --- | --- |
| Vascular | 0.28 | 0.29 | 0.25 | 0.28 | 0.28 | 0.31 | 0.32 | 0.31 | 0.27 | 0.30 | 0.29 | 0.02 |
| Largest Size Group | 0.21 | 0.13 | 0.09 | 0.11 | 0.17 | 0.13 | 0.12 | 0.14 | 0.14 | 0.16 | 0.14 | 0.03 |
| Pathology Final | 0.05 | 0.12 | 0.12 | 0.12 | 0.13 | 0.14 | 0.12 | 0.11 | 0.09 | 0.09 | 0.11 | 0.03 |
| Age | 0.15 | 0.10 | 0.11 | 0.10 | 0.10 | 0.11 | 0.09 | 0.10 | 0.07 | 0.08 | 0.10 | 0.02 |
| FH | 0.08 | 0.05 | 0.08 | 0.09 | 0.06 | 0.08 | 0.10 | 0.07 | 0.11 | 0.11 | 0.08 | 0.02 |
| Feeding Group | 0.04 | 0.04 | 0.11 | 0.06 | 0.05 | 0.04 | 0.03 | 0.06 | 0.07 | 0.05 | 0.05 | 0.02 |
| DCIS % Group | 0.04 | 0.08 | 0.05 | 0.06 | 0.05 | 0.04 | 0.05 | 0.06 | 0.04 | 0.03 | 0.05 | 0.01 |
| HER2 | 0.02 | 0.03 | 0.06 | 0.04 | 0.05 | 0.05 | 0.05 | 0.03 | 0.05 | 0.04 | 0.04 | 0.01 |
| Ki67% | 0.03 | 0.03 | 0.02 | 0.06 | 0.04 | 0.02 | 0.04 | 0.04 | 0.04 | 0.03 | 0.04 | 0.01 |
| Pregnancy Gropu | 0.02 | 0.04 | 0.04 | 0.03 | 0.05 | 0.03 | 0.04 | 0.03 | 0.04 | 0.03 | 0.03 | 0.01 |
| Side Group | 0.01 | 0.01 | 0.04 | 0.03 | 0.01 | 0.02 | 0.02 | 0.02 | 0.04 | 0.04 | 0.02 | 0.01 |
| First Pregnancy Group | 0.00 | 0.02 | 0.01 | 0.01 | 0.01 | 0.02 | 0.03 | 0.03 | 0.04 | 0.03 | 0.02 | 0.01 |
| ER | 0.06 | 0.03 | 0.00 | 0.01 | 0.00 | 0.00 | 0.01 | 0.00 | 0.00 | 0.01 | 0.01 | 0.02 |
| PR | 0.01 | 0.02 | 0.00 | 0.00 | 0.00 | 0.00 | 0.00 | 0.00 | 0.00 | 0.00 | 0.00 | 0.01 |

DCIS: Ductal carcinoma in situ; ER: Estrogen Receptor; FH: Family history; Gp: Group; PR: Progestron receptor;

**Table S3.** Feature Importance during each fold for Logistic Regression Model (sorted largest to smallest)

|  | Fold 1 | Fold 2 | Fold 3 | Fold 4 | Fold 5 | Fold 6 | Fold 7 | Fold 8 | Fold 9 | Fold 10 | Average | STD |
| --- | --- | --- | --- | --- | --- | --- | --- | --- | --- | --- | --- | --- |
| Vascular | 2.03 | 1.95 | 1.96 | 1.92 | 1.99 | 1.86 | 1.96 | 1.99 | 1.86 | 1.83 | 1.94 | 0.07 |
| Side Group | 0.98 | 0.84 | 0.74 | 0.68 | 0.78 | 0.74 | 0.68 | 0.98 | 0.76 | 0.72 | 0.79 | 0.11 |
| Largest Size Group | 0.59 | 0.53 | 0.58 | 0.60 | 0.58 | 0.53 | 0.51 | 0.60 | 0.72 | 0.70 | 0.60 | 0.07 |
| Pregnancy Gropu | 0.49 | 0.62 | 0.56 | 0.56 | 0.45 | 0.63 | 0.56 | 0.52 | 0.53 | 0.53 | 0.54 | 0.05 |
| PR | 0.47 | 0.45 | 0.30 | 0.36 | 0.47 | 0.35 | 0.37 | 0.44 | 0.47 | 0.39 | 0.41 | 0.06 |
| Feeding Group | 0.30 | 0.29 | 0.31 | 0.27 | 0.19 | 0.33 | 0.25 | 0.20 | 0.26 | 0.34 | **0.27** | **0.05** |
| Pathology Final | 0.22 | 0.17 | 0.13 | 0.13 | 0.19 | 0.15 | 0.20 | 0.16 | 0.12 | 0.15 | 0.16 | 0.03 |
| HER2 | 0.10 | 0.20 | 0.19 | 0.21 | 0.08 | 0.03 | 0.14 | 0.11 | 0.11 | 0.07 | 0.12 | 0.06 |
| ER | 0.05 | 0.08 | 0.17 | 0.16 | 0.06 | 0.14 | 0.12 | 0.15 | 0.08 | 0.13 | 0.11 | 0.04 |
| FH | 0.09 | 0.11 | 0.12 | 0.11 | 0.08 | 0.06 | 0.08 | 0.16 | 0.11 | 0.17 | 0.11 | 0.03 |
| DCIS % Group | 0.08 | 0.05 | 0.10 | 0.02 | 0.09 | 0.09 | 0.12 | 0.11 | 0.13 | 0.13 | 0.09 | 0.04 |
| First Pregnancy Group | 0.07 | 0.01 | 0.06 | 0.08 | 0.00 | 0.07 | 0.02 | 0.01 | 0.02 | 0.09 | 0.04 | 0.03 |
| Age | 0.02 | 0.02 | 0.02 | 0.02 | 0.02 | 0.02 | 0.03 | 0.02 | 0.02 | 0.02 | 0.02 | 0.00 |
| Ki67% | 0.00 | 0.00 | 0.00 | 0.00 | 0.00 | 0.00 | 0.00 | 0.01 | 0.00 | 0.00 | 0.00 | 0.00 |

DCIS: Ductal carcinoma in situ; ER: Estrogen Receptor; FH: Family history; Gp: Group; PR: Progestron receptor;


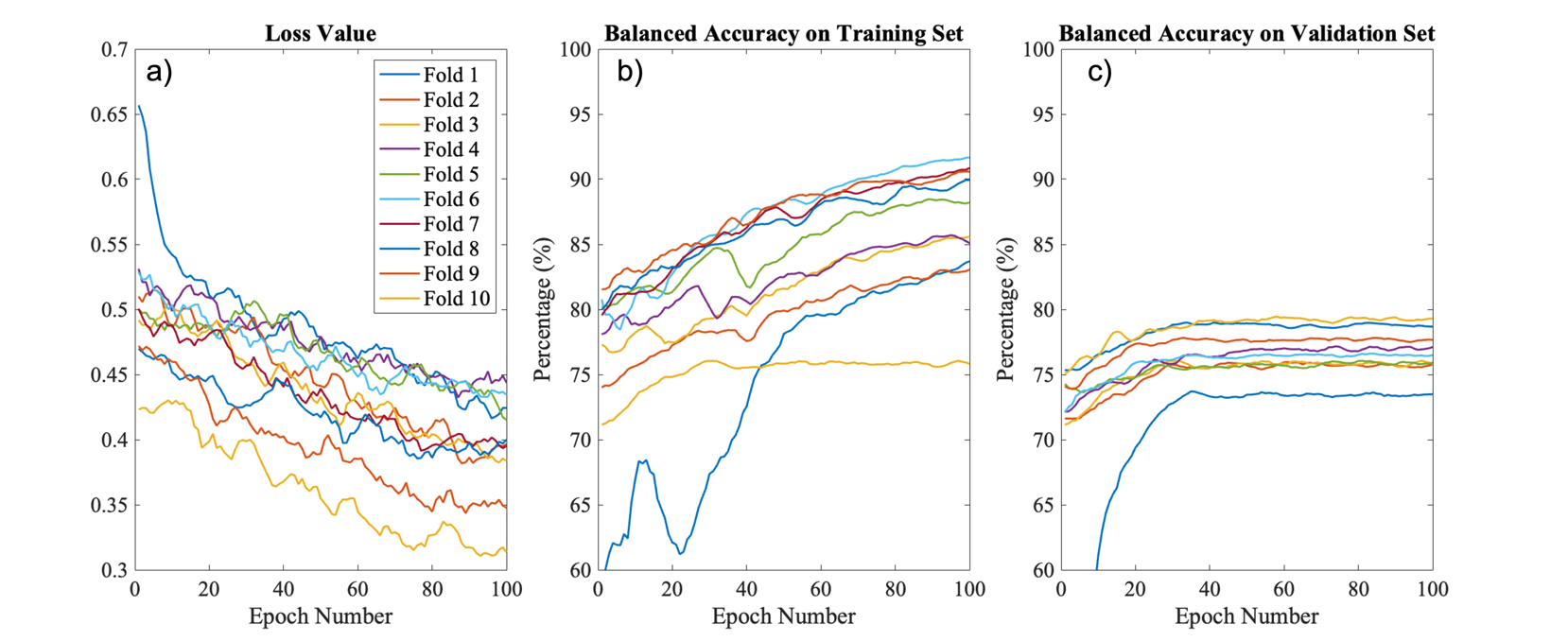


**Figure S1**. The trends observed a) loss function value, b) Balanced Accuracy in test set during training and c) Balanced Accuracy in validation set during training at each epoch for all folds using the TabNet Model.
